# Biological Basis of miRNA Action when Their Targets Are Located in Human Protein Coding Region

**DOI:** 10.1371/journal.pone.0063403

**Published:** 2013-05-06

**Authors:** Wanjun Gu, Xiaofei Wang, Chuanying Zhai, Tong Zhou, Xueying Xie

**Affiliations:** 1 Research Center of Learning Sciences, Southeast University, Nanjing, Jiangsu, China; 2 Institute for Personalized Respiratory Medicine, The University of Illinois at Chicago, Chicago, Illinois, United States of America; 3 Section of Pulmonary, Critical Care, Sleep & Allergy, Department of Medicine, The University of Illinois at Chicago, Chicago, Illinois, United States of America; University of Frankfurt - University Hospital Frankfurt, Germany

## Abstract

Recent analyses have revealed many functional microRNA (miRNA) targets in mammalian protein coding regions. But, the mechanisms that ensure miRNA function when their target sites are located in protein coding regions of mammalian mRNA transcripts are largely unknown. In this paper, we investigate some potential biological factors, such as target site accessibility and local translation efficiency. We computationally analyze these two factors using experimentally identified miRNA targets in human protein coding region. We find site accessibility is significantly increased in miRNA target region to facilitate miRNA binding. At the mean time, local translation efficiency is also selectively decreased near miRNA target region. GC-poor codons are preferred in the flank region of miRNA target sites to ease the access of miRNA targets. Within-genome analysis shows substantial variations of site accessibility and local translation efficiency among different miRNA targets in the genome. Further analyses suggest target gene’s GC content and conservation level could explain some of the differences in site accessibility. On the other hand, target gene’s functional importance and conservation level can affect local translation efficiency near miRNA target region. We hence propose both site accessibility and local translation efficiency are important in miRNA action when miRNA target sites are located in mammalian protein coding regions.

## Introduction

miRNAs are a class of small non-coding RNAs that regulate gene expression in post-transcriptional stage [1]. In the process of miRNA action, miRNAs are first specifically bound to their target sites in mRNA transcripts [1]. After binding, targeted mRNA transcripts can be silenced by several mechanisms, such as gene silencing, translational repression and mRNA degradation [2]. In plants, most miRNA target sites are located in protein coding region of target gene. Unlike plants, animal miRNAs have their target sites mostly in 3′ untranslated region (3′-UTR) of mRNA transcripts [1]. Two recent studies have investigated the reason why mammalian miRNA target sites are restricted to the 3′-UTR of mRNA transcripts [3], [4]. They have suggested active mRNA translation may impede miRNA association with target mRNAs in mammalian genomes [3], [4]. But, increasing evidences have confirmed many functional miRNA target sites are located in protein coding region of mammalian mRNA transcripts as well [5]–[12]. Notably, genome-wide analysis of Argonaute (AGO) HITS-CLIP data [13] and PAR-CLIP data [14] have revealed almost half AGO binding sites are located in human protein coding region. Given the prevalence of miRNA target sites occurred in mammalian protein coding sequences, it is important to investigate biological factors that may affect miRNA action when their targets are located in mammalian protein coding regions.

Comparative genomic analyses have been successfully used in identifying important factors of miRNA action [1], [15]. For example, miRNA genes are evolutionary conserved between species [1], [16] and within species [17]. At the same time, nucleotides in miRNA target region are under negative selection as well [18], [19]. These have implicated the importance of sequence conservation for proper miRNA function [1]. Comparing with miRNA target sites that are located in 3′-UTR of mammalian mRNA transcripts, those in mammalian protein coding region still need code amino acid information of their translated proteins. Previous studies have suggested the degeneracy of genetic code enables DNA sequences to code extra regulatory information as well as amino acid sequences [20]. Biased usage of synonymous codons have been related to many biological processes, such as DNA stability [21], nucleosome positioning [22], mRNA stability [23]–[25], mRNA splicing [26], [27], nonsense mediated mRNA decay [28], translation initiation [29]–[31], translation elongation [32]–[36] and co-translational protein folding [37], [38]. Additionally, many studies have suggested synonymous codon choices near miRNA target sites are related to miRNA function in mammalian genomes. For example, synonymous substitution rate in miRNA target region is reduced in some mammalian genomes [39]–[42]. Tay *et al.* [12] have found silent mutations occurred in miRNA target sites can eliminate miRNA activity in mouse. Brest *et al.* [43] have suggested that human Crohn’s disease is caused by a synonymous mutation at the binding site of miR-196 in *IRGM*. Hence, analysis of synonymous codon usage in the flank region of miRNA target sites should be able to gain some insights of miRNA action.

In this paper, we analyzed the usage of synonymous codons in the flank region of miRNA target sites that are located in human protein coding sequences. We chose human as the example of mammalian species, since many functional miRNA targets have been experimentally identified in human protein coding region [14]. We considered site accessibility and local translation efficiency as two possible biological determinants of miRNA action when their target sites are located in human protein coding region. Site accessibility is one of the most important factors that affect miRNA binding when miRNA target sites are located in 3′-UTR of mammalian mRNA transcripts [44], [45]. On the other hand, local translation efficiency around miRNA target region is particularly crucial for those targets located in mammalian protein coding region [3], [4]. We hypothesized that if site accessibility and local translation efficiency are important in miRNA action, they should be selectively varied at synonymous codon sites that are in the flank region of miRNA target sites. We computed site accessibility and local translation efficiency for each miRNA target in human protein coding region. To estimate the selection pressure and its significance, we permuted mRNA sequences and assessed the deviation of local translation efficiency and site accessibility from random expectation. We addressed the following several problems: 1) is site accessibility important in miRNA action when their target sites are located in protein coding region of mammalian transcripts? 2) Is local translation efficiency near miRNA target region also important for proper miRNA function? 3) If site accessibility and/or local translation efficiency are selectively varied near miRNA target region, what are the factors that can affect the selection pressure?

## Results

### Site accessibility is selectively varied in the flank region of miRNA target sites


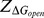
 measures the extent to which site accessibility deviates from random expectation. A negative 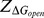 means that site accessibility is increased, and a positive 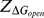 means it is decreased. We calculated 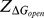 along mRNA sequences in sliding windows of 48 nucleotides in length. We started from the miRNA target region, which contains 21 nucleotides bound to miRNAs, 17 flank upstream nucleotides and 10 flank downstream nucleotides. We moved the sliding window both upward and downward along the mRNA sequences in a step of 48 nucleotides. We calculated 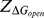 values in 13 consecutive windows for each miRNA target. For each window, we calculated a genomic mean value of 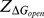 by averaging 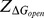 values over all miRNA targets in the human genome.

**Figure 1:**
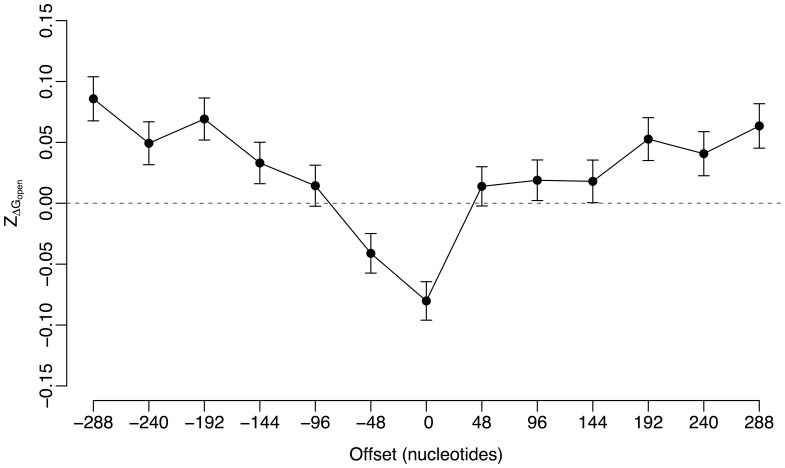
The mean and standard error of 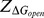 of each sliding window near miRNA target region in the human genome.

Figure 1 shows the genomic mean value of 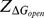 for all 13 consecutive windows in human. We observed a significant negative deviation of 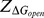 from zero (*t*-test, P<<10^−6^) in the central window. The negative values of 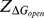 in the central window suggest selection for increased site accessibility in miRNA target region. When the sliding window moved upward or downward along the mRNA sequence, 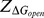 values increase quickly and most windows have significant positive mean 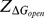 values. The positive 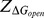 values in these windows suggest decreased site accessibility is generally preferred in mRNA segments other than the miRNA target region. In following analysis, we used 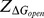 in the central window to represent the selection signal of site accessibility in miRNA target region.

### Translation efficiency is also selectively varied near miRNA target region


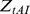
 measures the deviation of local translation efficiency from random expectation. A negative 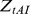 value means local translation efficiency is reduced, and a positive 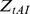 value means it is increased. We calculated 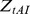 along mRNA sequences using a sliding window of nine codons (27 nucleotides) in length. We started from a window that is right downstream of miRNA target sites, and moved the window upward and downward along the mRNA sequence at a step of nine codons. We calculated 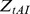 in 29 consecutive windows for each miRNA target region. For each window, we calculated a genomic mean 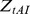 by averaging 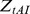 values over all miRNA target regions.

**Figure 2:**
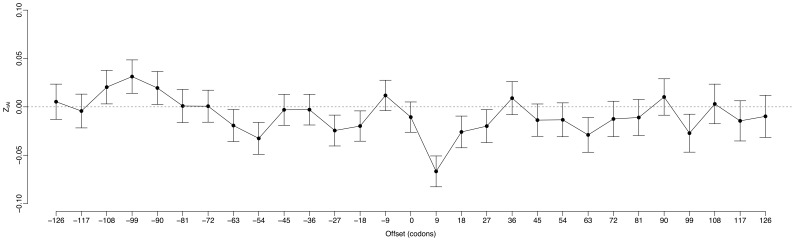
The mean and standard error of 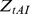 of each sliding window near miRNA target region in the human genome.

Figure 2 shows mean 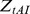 values in all 29 consecutive windows. We observed a significant negative deviation of 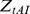 value from zero (*t*-test, P<<10^−4^) in a window that is nine codons downstream from the start point. The negative 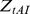 values in this window suggest decreased translation efficiency is selectively preferred near miRNA target sites. We did not observe any significant deviation of 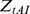 values from zero in other windows. This suggests reduced translation efficiency is only selectively preferred in the flank region of miRNA target sites. We used 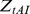 values in the window nine codons downstream of the start point to represent selection signal of local translation efficiency for following analysis.

### GC-poor codons are preferred in miRNA target region

**Figure 3:**
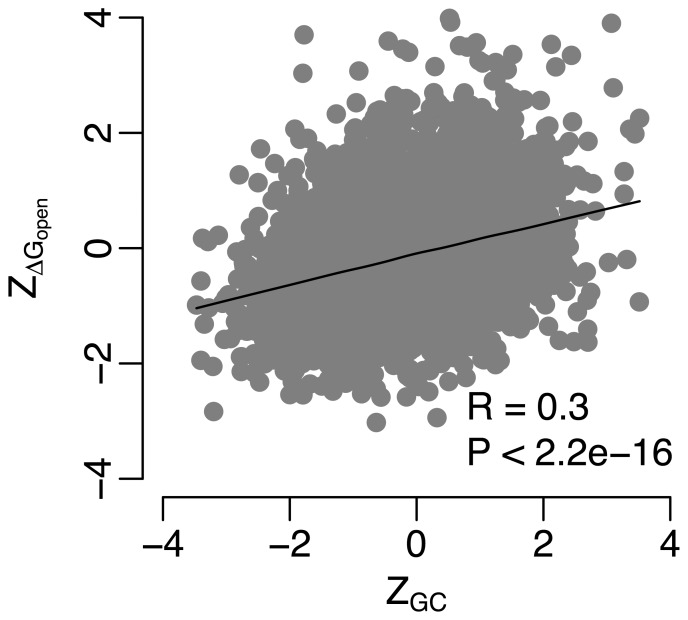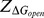 in miRNA target region as a function of 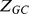 in that region. Each point represents a miRNA target in human protein coding sequences.

We observed site accessibility tended to increase in miRNA target region (Figure 1). Site accessibility is mainly determined by RNA secondary structure near miRNA target region. We calculated *Z*-score of local RNA secondary structure, 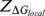, in all 13 sliding windows as we did in site accessibility analysis. We also observed decreased RNA stability in miRNA target region (Figure S1). 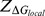 in miRNA target region is correlated well to 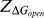 in that region (Pearson’s product-moment correlation = −0.3604834, P<<10^−15^; Figure S2). When looking into GC content in miRNA target region, we also observed a significant negative deviation of 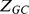 values from zero (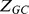 = −0.106+/−0.034; *t*-test, P = 1.3*10^−9^). A negative 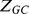 value in miRNA target region suggests GC-poor codons are selectively preferred in that region. When comparing 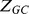 and 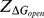 of each miRNA target, we observed a significant positive correlation (Pearson’s product-moment correlation = 0.3, P<<10^−15^; Figure 3). We also observed a significant negative correlation (Pearson’s product-moment orrelation = 0.25, P<<10^−15^; Figure S3) between 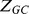 and 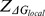 in miRNA target region. This suggests GC-poor codons are selectively preferred in miRNA target region for increased site accessibility by loosing RNA structure in that region.

### Factors that influence the selection pressure among miRNA target regions

In previous sections, we considered the mean 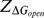 and 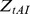 over all miRNA targets in protein coding region of the human genome. But, we observed substantial variations of site accessibility and translation efficiency among different miRNA targets in the genome (Figures 1 and 2). We next investigated the factors that may affect site accessibility and local translation efficiency of miRNA targets.

**Figure 4:**
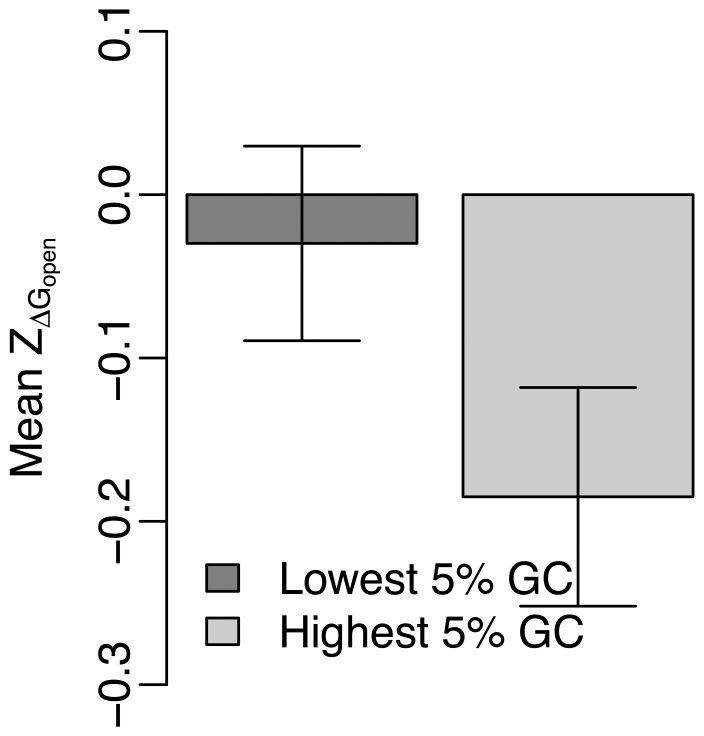
Comparison of the mean 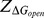 between miRNA targets in genes with the highest 5% and the lowest 5% GC content.

We first considered target gene’s GC content. We selected miRNA targets in genes with the highest 5% and the lowest 5% GC content, and compared the mean 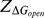 and 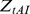 of miRNA targets in these two groups. We observed 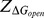 values of miRNA targets in GC-rich genes were significantly smaller than those in GC-poor genes (Welch Two Sample *t*-test, P = 0.04; Figure 4). This suggests miRNA targets in GC-rich genes are under higher selection pressures to increase site accessibility. But, 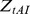 values of miRNA targets in genes with the highest 5% GC content and those with the lowest 5% GC content are not statistically different (Welch Two Sample *t*-test, *P* = 0.42; Figure S4).

**Figure 5:**
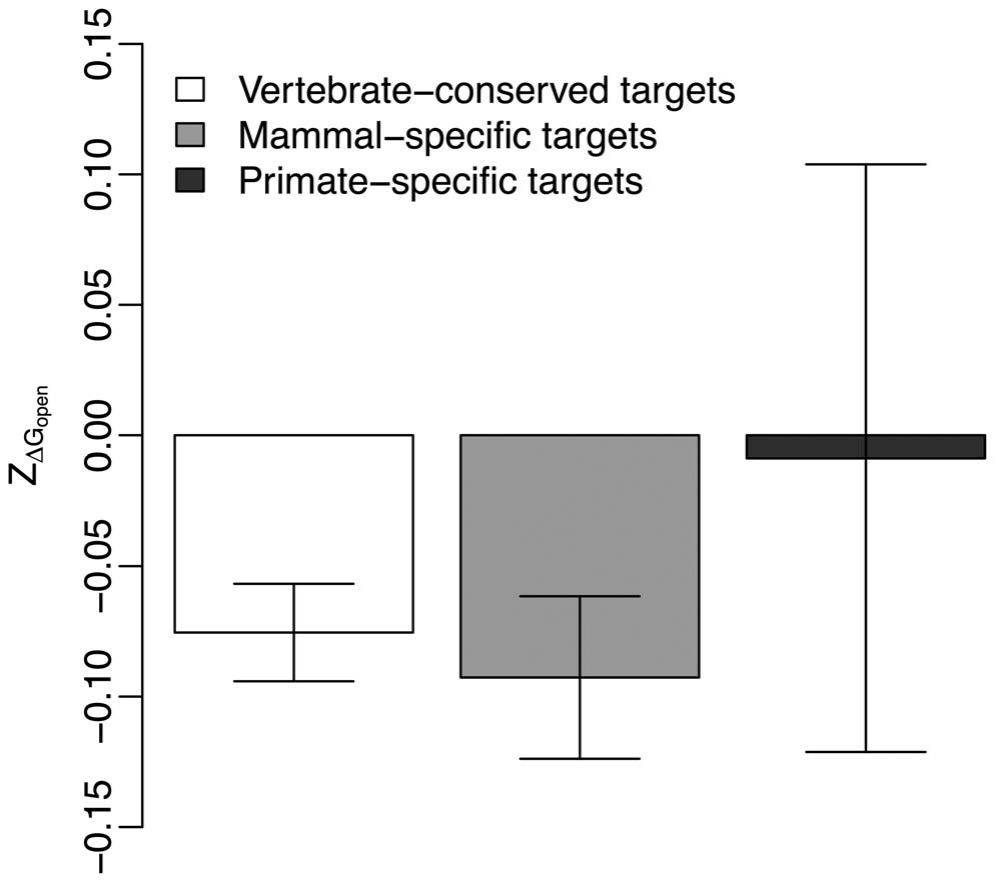
Comparison of the mean 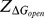 between miRNA targets in genes at different conservation levels.

**Figure 6:**
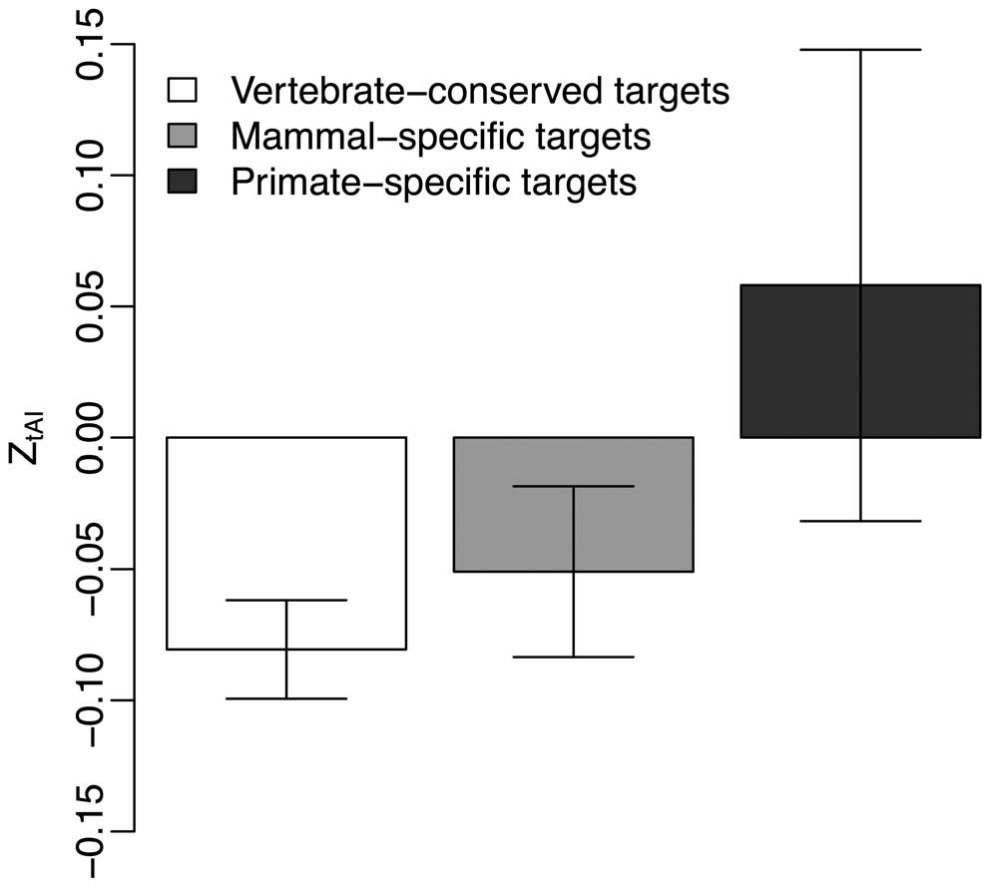
Comparison of the mean 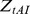 between miRNA targets in genes at different conservation levels.

Next we considered the conservation level of miRNA target gene. We separated miRNA targets into three groups based on gene conservation, including primate-specific targets, mammal-specific targets and vertebrate-conserved targets. We compared 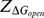 and 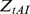 values of miRNA targets in these three groups. Both 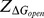 and 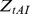 mean of mammal-specific and vertebrate-conserved targets are smaller than those in primate-specific targets (Figures 5 and 6). While 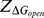 values of mammal-specific targets (*t*-test, P<<10^−4^) and vertebrate-conserved targets (*t*-test, P = 0.003) are significantly deviated to zero, 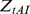 values of mammal-specific targets (*t*-test, P<<10^−4^) and vertebrate-conserved targets (*t*-test, P = 0.012) are also significantly deviated to zero. But, neither 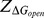 (*t*-test, P = 0.938) nor 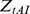 values (*t*-test, P = 0.519) in primate-specific targets have significant deviation to zero. This suggests selection of site accessibility and translation efficiency is more pronounced for miRNA targets with longer evolutionary history.

**Figure 7:**
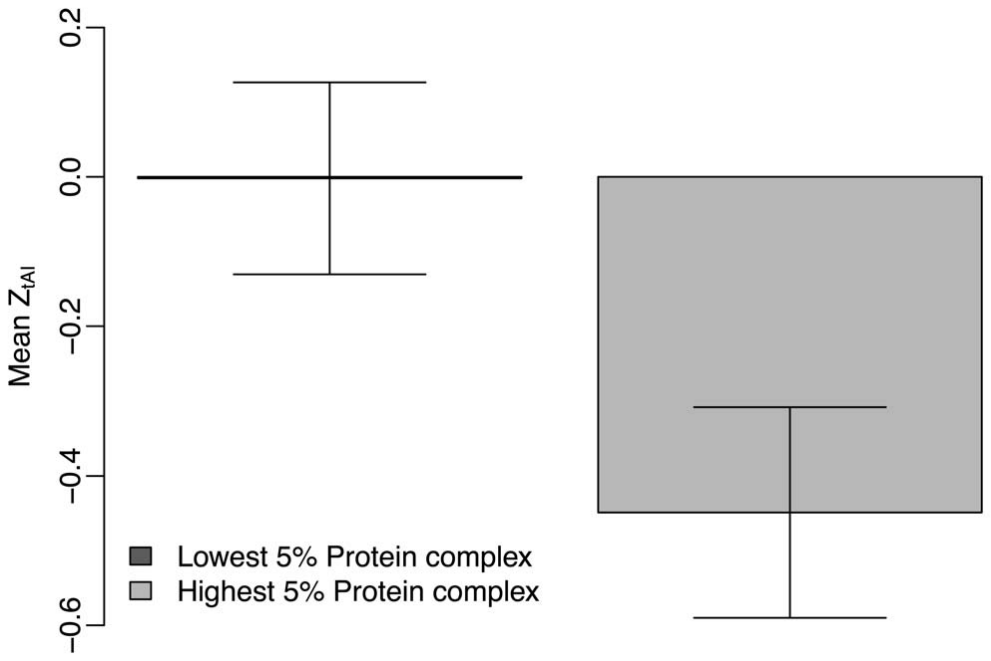
Comparison of the mean 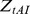 between miRNA targets in genes with the highest 5% and lowest 5% protein complex size.

We then considered the functional importance of target gene. We used the protein complex size to denote gene’s functional importance. We compared 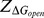 and 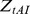 values of miRNA targets in genes with the highest 5% complex size to those with the lowest 5% complex size. The mean 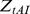 value of miRNA targets in genes with higher functionality is smaller than that of miRNA targets in genes with less functionality (Figure 7). We did not observe any significant difference between 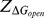 values of miRNA targets in genes with different functional importance (Figure S5).

We finally considered the expression level and codon bias of target gene. We used *ENC* (Effective Number of Codons) to measure a gene’s codon usage bias [46]. The higher a gene’s codon usage bias, the lower the gene’s *ENC*. We compared 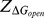 and 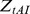 values of miRNA targets in genes the highest 5% expression level to those with the lowest 5% expression level. We also compared 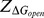 and 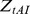 values of miRNA targets in genes with the highest 5% *ENC* to those with the lowest 5% *ENC*. But, we did not observe any significant difference in these comparisons (Figures S6, S7, S8 and S9).

## Discussion

To investigate the biological basis of miRNA action when miRNA targets are located in protein coding region of mammalian transcripts, we have performed a genome scale analysis of site accessibility and translation efficiency near miRNA target region in the human genome. We have found both site accessibility and translation efficiency is selectively varied in the flank region of miRNA target sites (Figures 1 and 2). By focusing on *Z*-scores relative to the random expectation, our method has excluded some biases such as amino acid content or preferred codon usage that may cause the signal. Site accessibility in miRNA target region tends to be higher in real mRNA sequence than that expected from the permutated mRNA sequences (Figure 1). Our results are comparable to the observations made in some previous studies 44,45. They had found site accessibility was the key factor in determining miRNA activity when miRNA target sites are located in 3′-UTR of human transcripts. Another two experimental studies had found active mRNA translation could impede miRNA association with target mRNAs in mammalian genomes [3], [4]. When rare codons are introduced in the upstream region of miRNA target sites, miRNA activity could be restored [3]. In our results, we have found translation efficiency is decreased in the flank region of miRNA target sites (Figure 2). This confirms the importance of local translation efficiency in miRNA action for miRNA targets occurred in mammalian protein coding region. We have not observed any significant correlation between site accessibility and translation efficiency in the flank region of miRNA target sites (Pearson’s product-moment correlation = 0.02, P = 0.27; Figure S10). Hence, site accessibility and local translation efficiency may be two independent factors in miRNA action when miRNA targets are located in mammalian protein coding regions.

In previous analysis, we had found site accessibility, but not local translation efficiency, was related to miRNA action when miRNA targets are located in protein coding region of plant genes [47], [48]. This suggests site accessibility is a much general factor that affects miRNA action for different kinds of miRNA targets [44], [45], [47]. Comparing with site accessibility, local translation efficiency is only effective when miRNA targets are located in mammalian protein coding region [3], [4], [48]. Is it possible that the selection signal for reduced translation efficiency near miRNA target sites is an artifact caused by our method? To eliminate this possibility, for each miRNA target, we randomly picked a region of 21 nucleotides in length from the same mRNA sequence. We combined all these randomly selected gene regions as a dataset of randomized miRNA targets. We replicated our analysis of translation efficiency on all randomized miRNA targets. We did not observe any selection signal of reduced translation efficiency in all sliding windows (Figure S11). This implies that reduced translation efficiency near miRNA target region we have observed Figure 2 is a real signal. The reason why reduced translation efficiency is only important for miRNA targets in mammalian protein coding region is unknown. Some analysis had suggested most animal miRNAs regulated their target genes by translational repression, while plant miRNAs regulated their target genes mainly by RNA degradation [1], [2]. This difference may explain the different importance of local translation efficiency in miRNA action between plants and mammals.

In our results, the region that tends to increase site accessibility is miRNA target region, which includes miRNA target sites, 17 flank upstream nucleotides and 10 downstream nucleotides (Figure 1). This region is the same as the one that we had observed in four plant genomes [29], and is comparable to those had been identified from experimental data [44], [45]. We have observed that 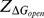 value in most other windows is positive (Figure 1). This implies site accessibility in mRNA sequences is generally decreased. This is mainly caused by much tighter RNA structure in mRNA sequences (Figure S1). The latter result is comparable to the observations made by several previous studies that the overall mRNA stability is selectively increased in human [49], [50].

We have observed that the region with decreased translation efficiency is located downstream of miRNA target sites (Figure 2). This region is different from the one suggested by Gu *et al.* [3]. They had employed model reporter constructs by introducing mutations into stop codons located upstream of miRNA target sites, which allowed translation to proceed through miRNA target sites [3]. They had found active translation could totally impede miRNA regulation. But, miRNA regulation can be restored when nine consecutive rare codons are introduced right upstream of miRNA target sites. In contrast, Lin *et al.* [4] had analyzed two naturally occurred miRNA target sites in viral protein coding sequences. They had suggested translation could modestly decrease miRNA regulation of those two naturally occurred miRNA targets. They had found the distribution of rare codons in the upstream region of those two miRNA targets is same as that of random sequences [4]. Our results are largely comparable to that observed in Lin *et al.* [4]. For naturally occurred miRNA targets in human protein coding sequences, we have observed reduced translation efficiency is selectively preferred near miRNA target region. But, translation efficiency in the region right upstream of miRNA target sites is not selectively varied. Instead, reduced translation efficiency has been observed in a nearby region located downstream of the miRNA target sites. A possible explanation is naturally occurred miRNA targets may use two separate regions to facilitate miRNA binding. Since the region right upstream of miRNA target sites has already been used to increase site accessibility, they may use the region downstream of the miRNA target region to slow down translational process near miRNA target sites. These two layers of selection may act together in the flank region of miRNA targets to ensure proper miRNA activity. It will be interesting to set up some experiments to validate this possibility.

Our results have suggested that the conservation level of target gene is a general factor that affects both site accessibility and translation efficiency in miRNA target region. We have found 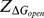 and 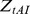 values are smaller in miRNA targets located in vertebrate-conserved genes and mammal-specific genes (Figures 5 and 6). But, miRNA targets in primate-specific genes have no obvious signal of selection for site accessibility and translation efficiency (Figures 5 and 6). These are consistent with the observations made by previous works [1], [17], [18], [51]. They had suggested purifying selection was smaller in younger miRNA targets than that in conserved miRNA targets.

We have found 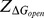 values of target regions in GC-rich genes are smaller than that in GC-poor genes (Figure 4). This finding can be explained by thermodynamic rules. Since GC pairs have three hydrogen bonds, GC-rich codons tend to fold in more stable RNA structures than AT-rich codons. Because loose RNA secondary structure is preferred in miRNA target region (Figure S1), GC-poor codons should be selectively preferred in miRNA target region to increase site accessibility, which is consistent with what we have observed in Figure 3. Assuming that selection targets the same higher site accessibility near all miRNA targets, we would expect that the increase of site accessibility is larger near miRNA targets located in GC-rich genes, simply because they start from a more-stable baseline. Whether selection actually targets the same higher site accessibility is not determined by our analysis. The actual larger decrease of 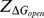 values for miRNA targets in GC-rich genes (Figure 4) implies this possibility.

In conclusion, we suggest site accessibility and translation efficiency have some effects on miRNA function when their targets are located in protein coding region of mammalian genes. Our results may help us better understand the process of miRNA action. It will also bring us important implications of protein coding sequence evolution in mammalian genome.

## Materials and Methods

### Data

Human PAR-CLIP data were downloaded from Supplementary Materials of Hafner *et al.* [14]. The PAR- CLIP data from Hafner *et al.* [14] contains genomic coordinates of DNA elements that were bound to human miRNAs in the experiment. Gene annotations of the human genome and mRNA sequences of all human transcripts were downloaded from Ensembl [52]. Overall, we extracted 4,344 miRNA targets that are located in human protein coding region.

To investigate the factors that may affect site accessibility and/or translation efficiency in the flank region of miRNA target sites, we considered the conservation level, the expression level and protein complex size of target gene. We downloaded a multiple alignment of 45 vertebrate genomes from UCSC genome server [53]. Using the 45-way alignment, we classified miRNA target genes into three groups (primate-specific genes, mammal-specific genes and vertebrate-conserved genes). We next downloaded expression data of human genes from Su *et al.* [54]. The expression level of each gene was quantified as the geometric mean of expression among different tissues. Finally, we downloaded protein complex data from the CORUM database [55]. For each gene, the complex size was measured as the number of proteins forming it and averaged across the number of complexes the gene participates.

### Site accessibility

Site accessibility represents the difficulty in opening a segment of mRNA sequences around miRNA target sites for their binding with RNA-induced silencing complex (RISCs) [15]. We used 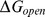 to measure site accessibility for each miRNA target. 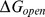 is the difference between free energy of the secondary structure of miRNA target region and free energy of the secondary structure in which miRNA target sites are unpaired [45]. 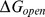 has been proved to be a good indicator of site accessibility [44], [45]. We used a window of 48 nucleotides, including 21 nucleotides directly bound by miRNAs, 17 flank upstream and 10 flank downstream nucleotides, to represent a miRNA target region. Previous studies have observed a significant correlation between 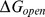 value of this region and miRNA activity [44], [45]. We calculated 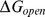 for each miRNA target using *RNAddG4* program in *PITA* package [45] with default parameter settings. At the same time, the folding energy of local RNA secondary structure, 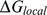, was also calculated for each miRNA target by RNAddG4 [45]. In *RNAddG4* [45], *RNAfold* [56] was used to calculate the free energy of RNA secondary structures. As suggested in previous studies [45], [57]–[60], we used a segment of mRNA sequences, rather than the full-length mRNAs, as the input sequences in calculating free energy of local RNA secondary structure. For each miRNA target region, the input mRNA segment includes 48 nucleotides in miRNA target region and additional 140 flank upstream and downstream nucleotides. This is based on the fact that the probability of base pairing when nucleotides are separated by more than 140 nucleotides was low (data not shown) and it can substantially reduce computational complexity.

### Translation efficiency

We used tRNA adaptation index (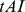) to quantify translation efficiency in the flank region of mRNA target sites. 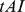 is a measure of codon adaptation to the tRNA abundance in genome [61]. 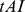 has been widely used to estimate translation efficiency in human [31], [61], [62]. We calculated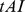 using codonR package [61], where tRNA copy numbers in the human genome were downloaded from the Genomic tRNA Database [63]. Since nine rare codons introduced in the upstream region of miRNA target sites are able to slow down local translational process and secure miRNA function [3], we calculated 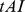 in a window of nine consecutive codons in the flank region of miRNA target sites as a measure of local translation efficiency for each miRNA target.

### mRNA randomization

We hypothesized that if site accessibility and/or local translation efficiency is important for miRNA action when miRNA target sites are located in human protein coding sequences, synonymous codons should be selected for site accessibility and/or local translation efficiency in human. To detect the selection signal on synonymous codons, we used a randomization method to compute the statistical deviation of site accessibility and/or translation efficiency between the real mRNA sequence and permuted sequences [29], [47]–[49]. We randomly shuffled synonymous codons among sites for each mRNA transcript, while keeping the encoded protein sequences, gene’s codon usage bias and gene’s GC composition the same. We did not shuffle the codons that are directly targeted by miRNAs during mRNA randomization, since those nucleotides are crucial for proper miRNA function. We generated 1,000 such permuted mRNA sequences for all mRNA transcripts with miRNA target sites in their protein-coding region.

### *Z*-score calculation

We used *Z*-score to determine the deviation of the real sequence from randomized sequences and estimate its statistical significance. For each miRNA target region, we calculated site accessibility in the real mRNA transcript 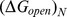 and each permutated mRNA sequence 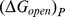. Then, we calculated *Z*-score of site accessibility (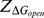) for each miRNA target region as formula 1.
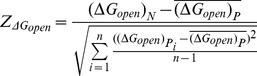
(1)


Here, 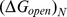 is site accessibility for the naturally occurring target region under consideration. 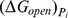 is site accessibility for the target region in 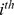 permuted sequence, and 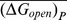 is the mean of 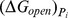 over all permuted sequences. The variable *n* represents the total number of permuted sequences, which is equal to 1,000 in our analysis.

Similarly, we calculated *Z*-score of local secondary structure (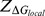), *Z*-score of local GC content (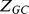) and *Z*-score of local translation efficiency (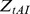) in the flank region of each miRNA target as formulas below.
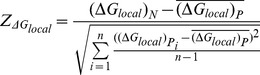
(2)

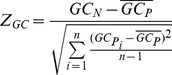
(3)

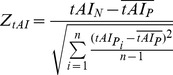
(4)


The definitions for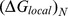, 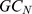, and 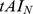 are analogous to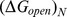, but refer to free energy of local RNA secondary structure, GC content and translation efficiency rather than to site accessibility. Similarly, the definitions for 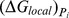, 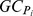, and 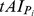 are analogous to 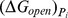, and the definitions for 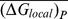, 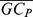, and 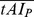 are analogous to_
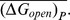
_

## Supporting Information

Figure S1
**The mean and standard error of 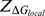 of each sliding window near miRNA target region in the human genome.**
(TIFF)

Figure S2

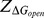
 **in miRNA target region as a function of 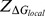 in that region.** Each point represents a miRNA target in human protein coding sequences.(TIFF)

Figure S3

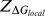
 **in miRNA target region as a function of 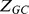 in that region.** Each point represents a miRNA target in human protein coding sequences.(TIFF)

Figure S4
**Comparison of the mean 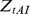 between miRNA targets in genes with the highest 5% and lowest 5% GC content.**
(TIFF)

Figure S5
**Comparison of the mean 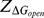 between miRNA targets in genes with the highest 5% and lowest 5% protein complex size.**
(TIFF)

Figure S6
**Comparison of the mean 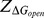 between miRNA targets in genes with the highest 5% and lowest 5% ENC.**
(TIFF)

Figure S7
**Comparison of the mean 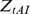 between miRNA targets in genes with the highest 5% and lowest 5% ENC.**
(TIFF)

Figure S8
**Comparison of the mean 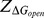 between miRNA targets in genes with the highest 5% and lowest 5% expression level.**
(TIFF)

Figure S9
**Comparison of the mean 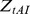 between miRNA targets in genes with the highest 5% and lowest 5% expression level.**
(TIFF)

Figure S10

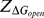
 **in miRNA target region as a function of 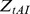 in the window that is nine codons downstream of miRNA target sites.** Each point represents a miRNA target in human protein coding sequences.(TIFF)

Figure S11
**The mean and standard error of 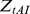 of each sliding window near randomized miRNA target region.**
(TIFF)
