# Supplementary figures and images for: Biological Basis of miRNA Action when Their Targets Are Located in Human Protein Coding Region
Source: PLoS One. 2013 May 6;8(5):e63403. doi: 10.1371/journal.pone.0063403 (PMC3646042; doi:10.1371/journal.pone.0063403)

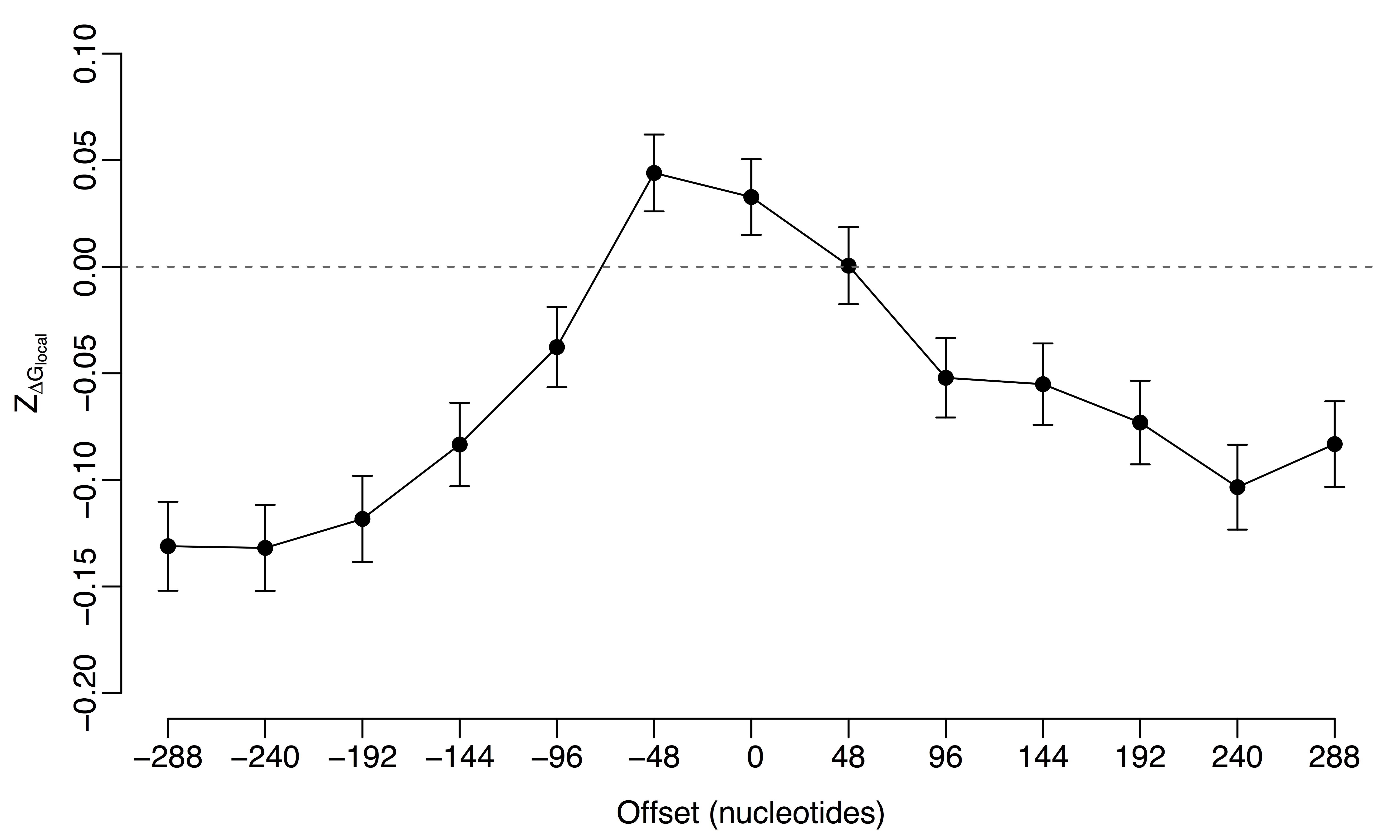

Supplement: Figure S1 — The mean and standard error of of each sliding window near miRNA target region in the human genome. (TIFF) [file pone.0063403.s001.tiff]

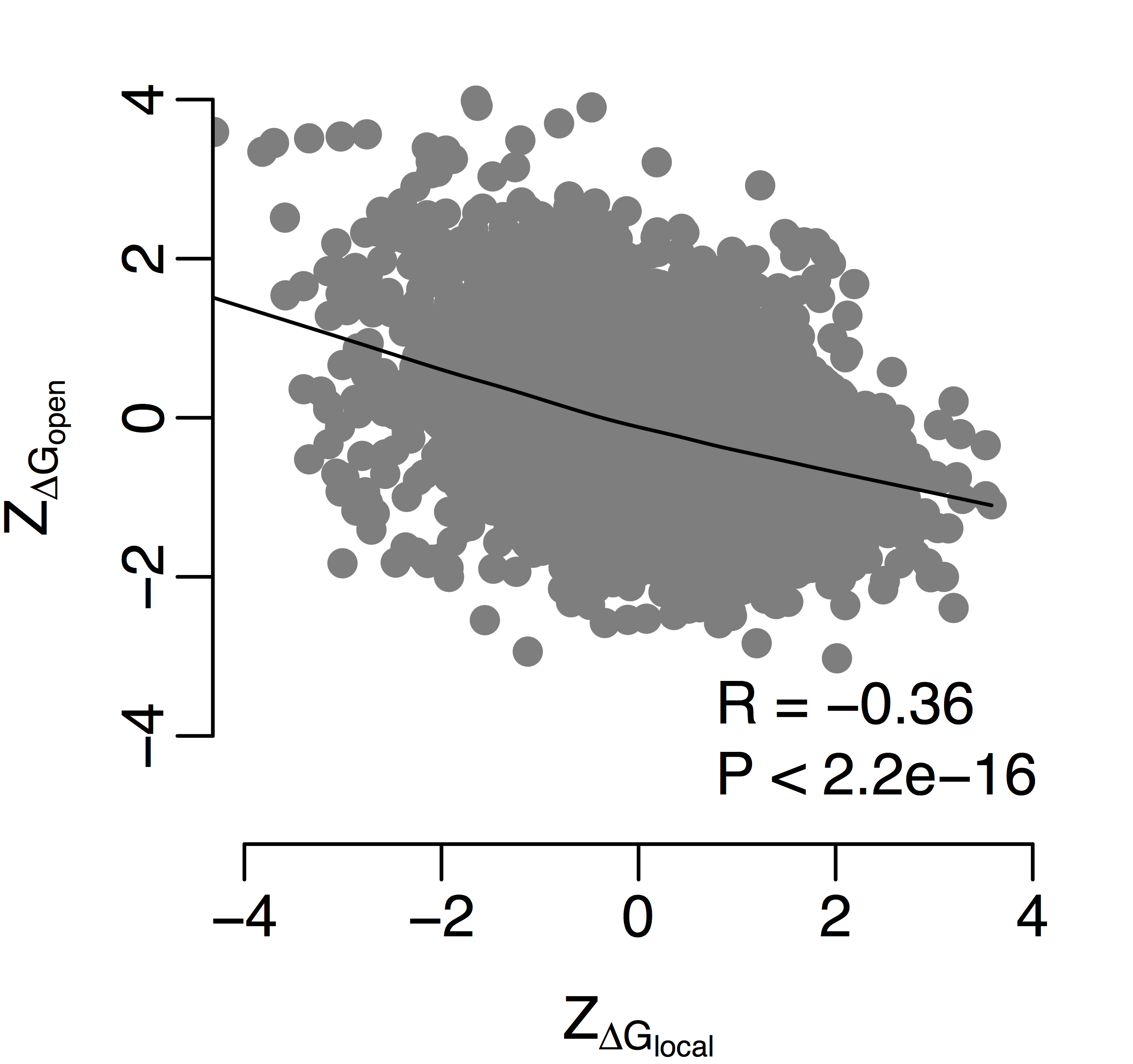

Supplement: Figure S2 — in miRNA target region as a function of in that region. Each point represents a miRNA target in human protein coding sequences. (TIFF) [file pone.0063403.s002.tiff]

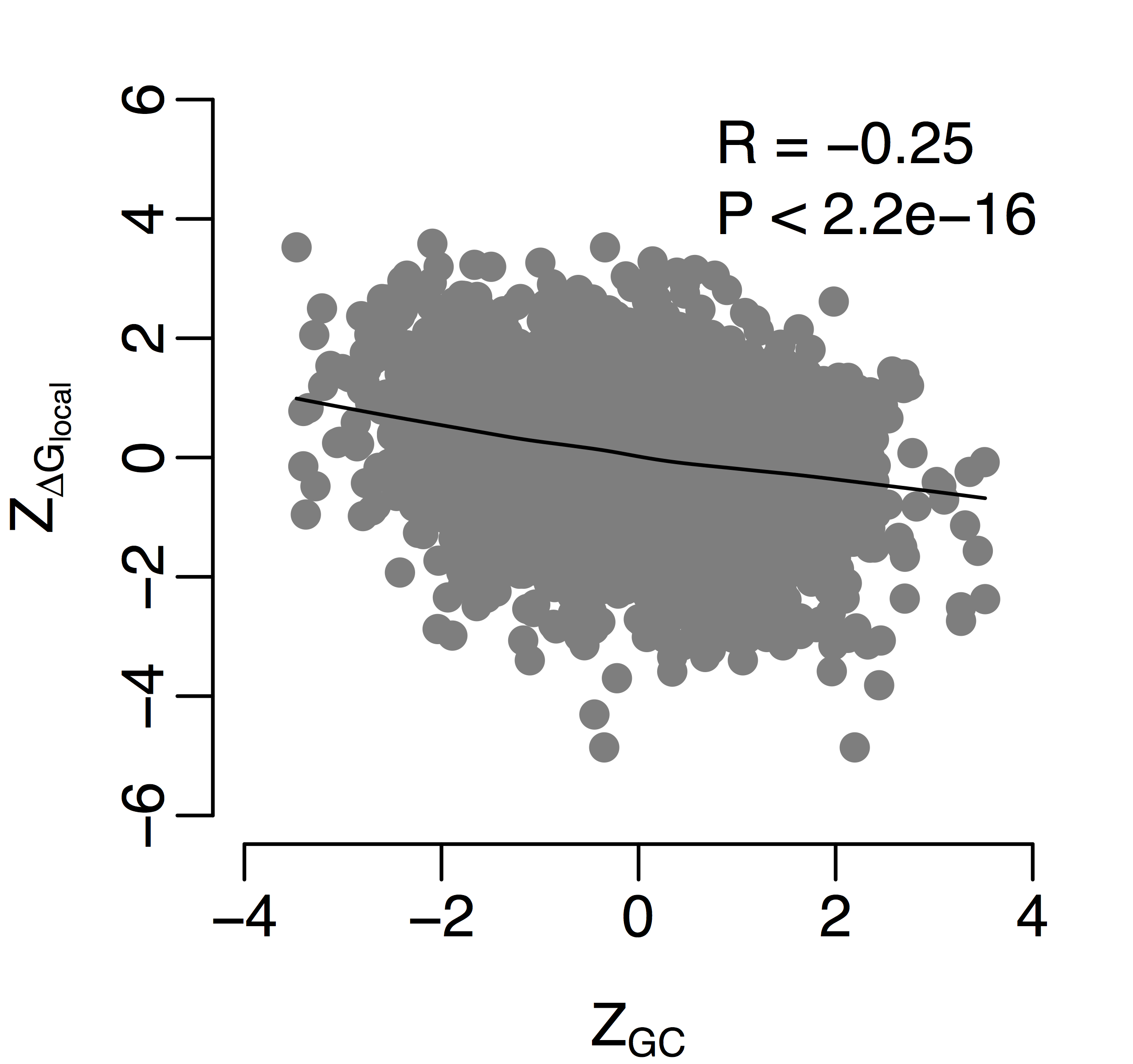

Supplement: Figure S3 — in miRNA target region as a function of in that region. Each point represents a miRNA target in human protein coding sequences. (TIFF) [file pone.0063403.s003.tiff]

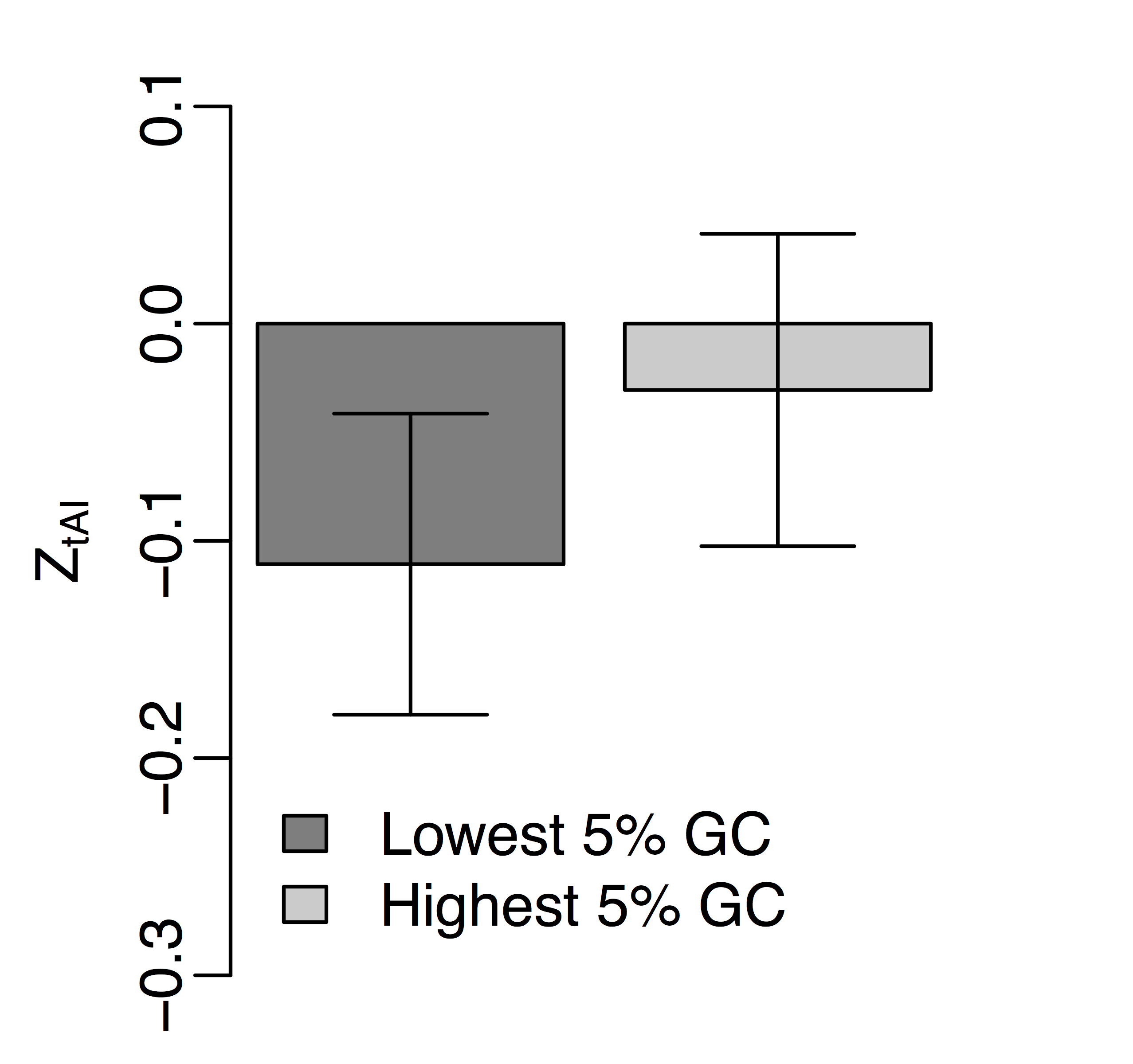

Supplement: Figure S4 — Comparison of the mean between miRNA targets in genes with the highest 5% and lowest 5% GC content. (TIFF) [file pone.0063403.s004.tiff]

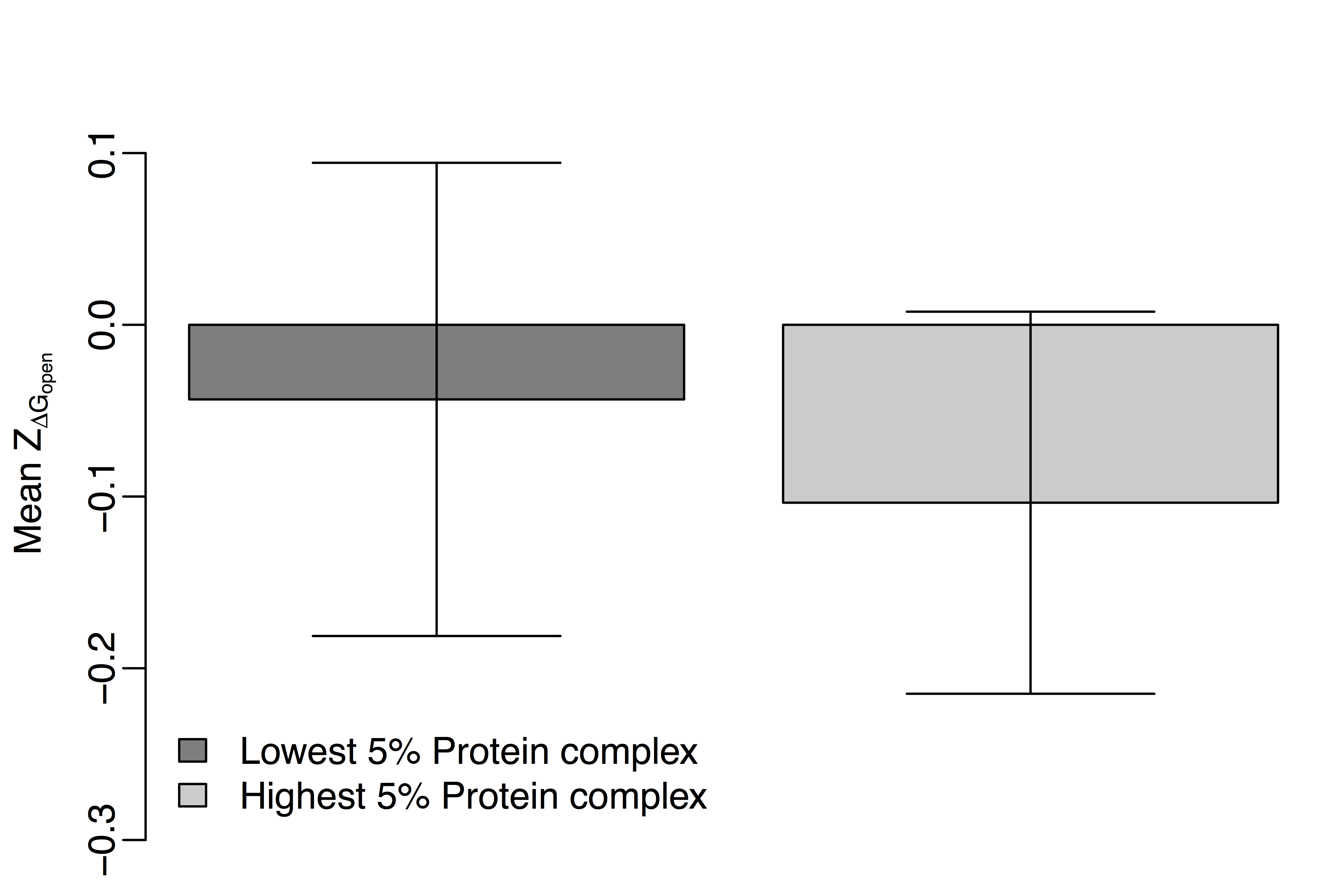

Supplement: Figure S5 — Comparison of the mean between miRNA targets in genes with the highest 5% and lowest 5% protein complex size. (TIFF) [file pone.0063403.s005.tiff]

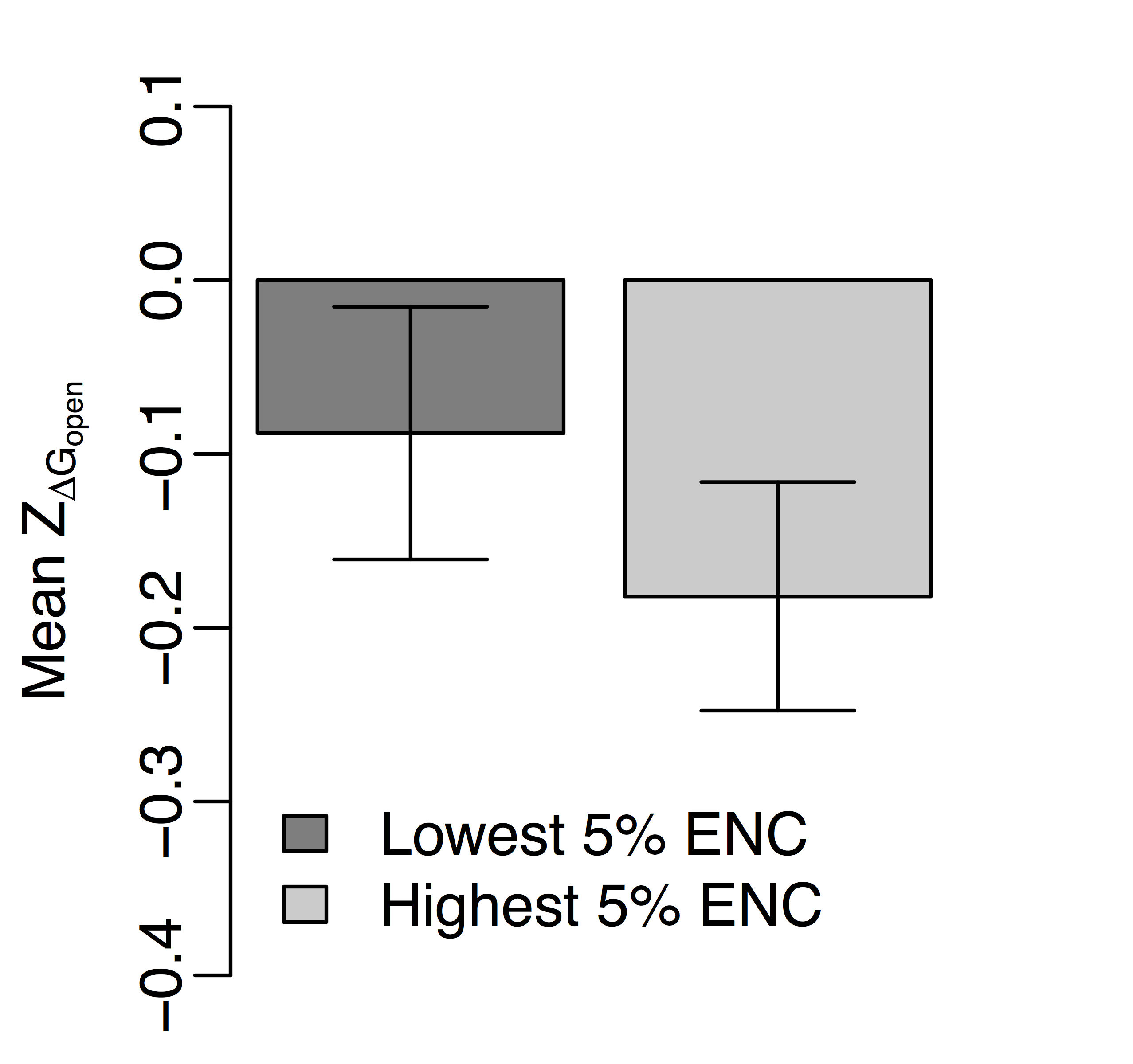

Supplement: Figure S6 — Comparison of the mean between miRNA targets in genes with the highest 5% and lowest 5% ENC. (TIFF) [file pone.0063403.s006.tiff]

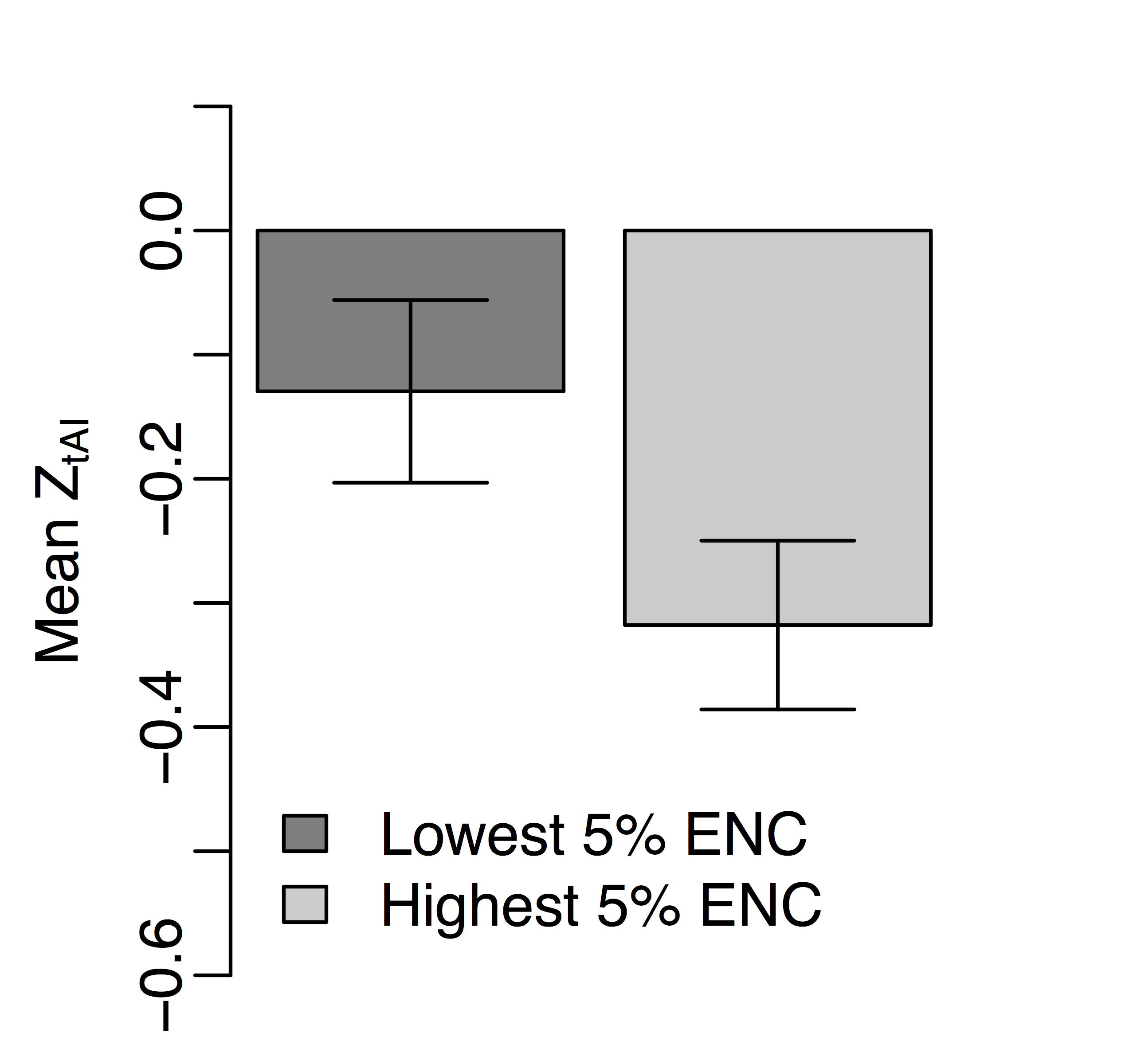

Supplement: Figure S7 — Comparison of the mean between miRNA targets in genes with the highest 5% and lowest 5% ENC. (TIFF) [file pone.0063403.s007.tiff]

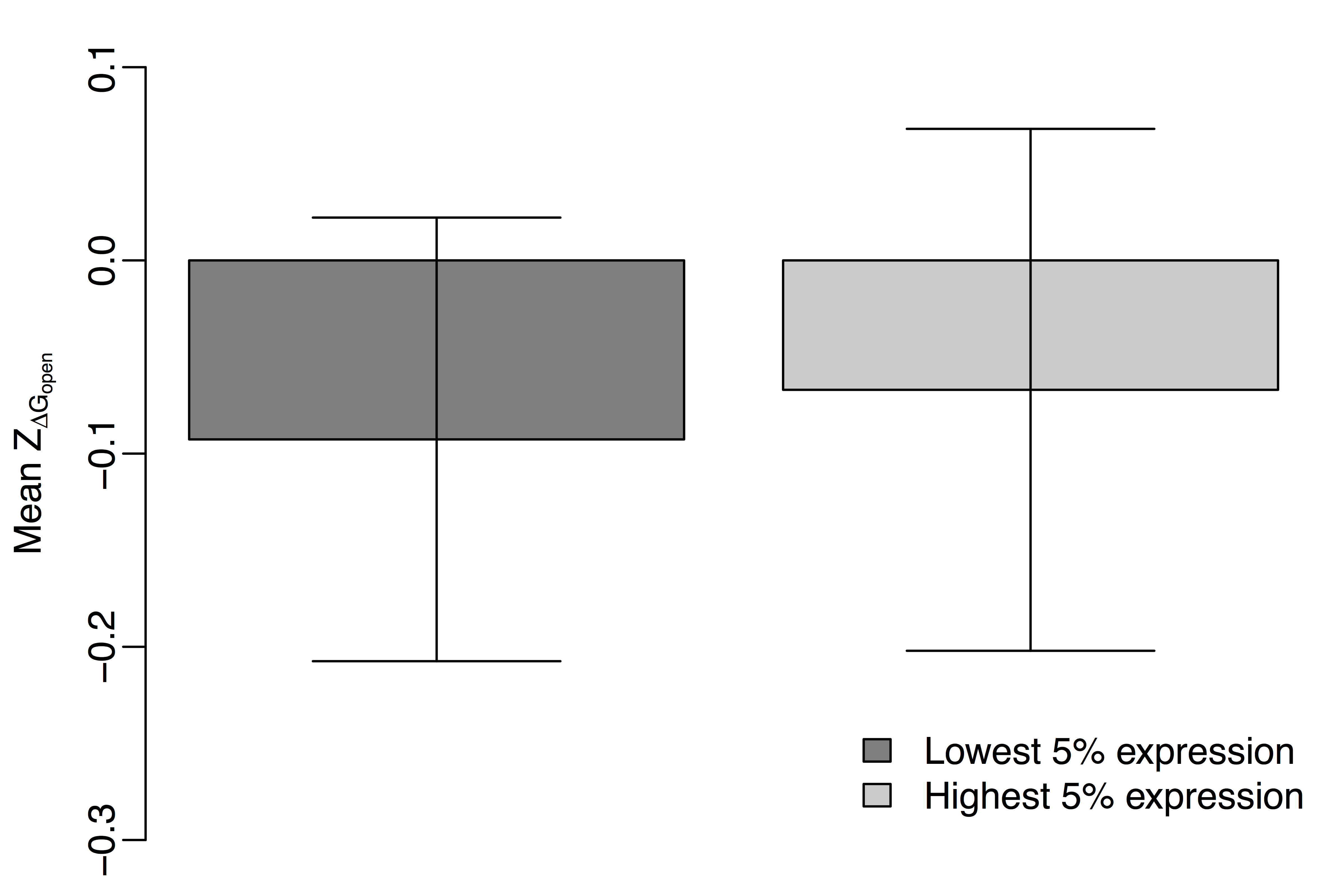

Supplement: Figure S8 — Comparison of the mean between miRNA targets in genes with the highest 5% and lowest 5% expression level. (TIFF) [file pone.0063403.s008.tiff]

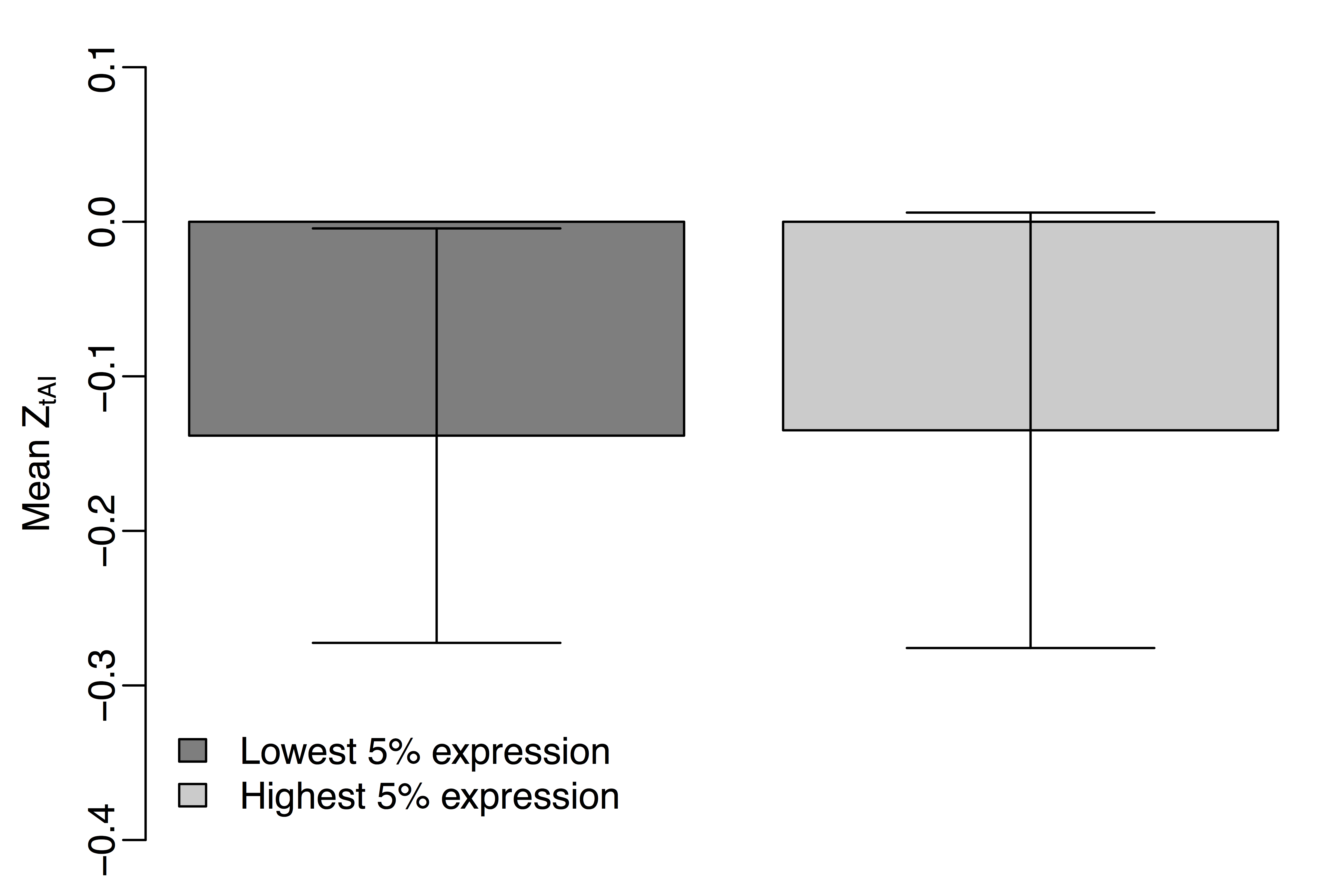

Supplement: Figure S9 — Comparison of the mean between miRNA targets in genes with the highest 5% and lowest 5% expression level. (TIFF) [file pone.0063403.s009.tiff]

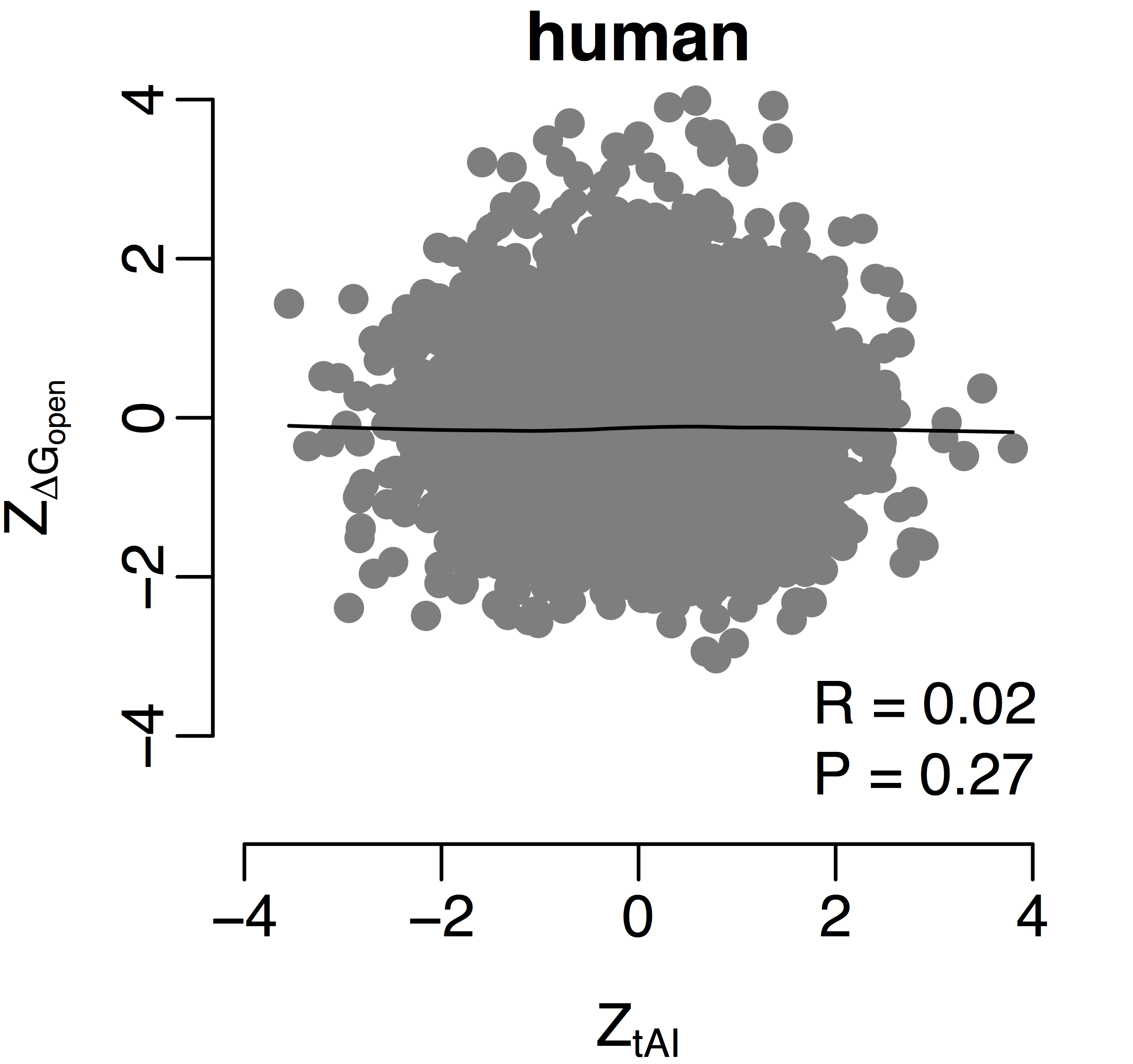

Supplement: Figure S10 — in miRNA target region as a function of in the window that is nine codons downstream of miRNA target sites. Each point represents a miRNA target in human protein coding sequences. (TIFF) [file pone.0063403.s010.tiff]

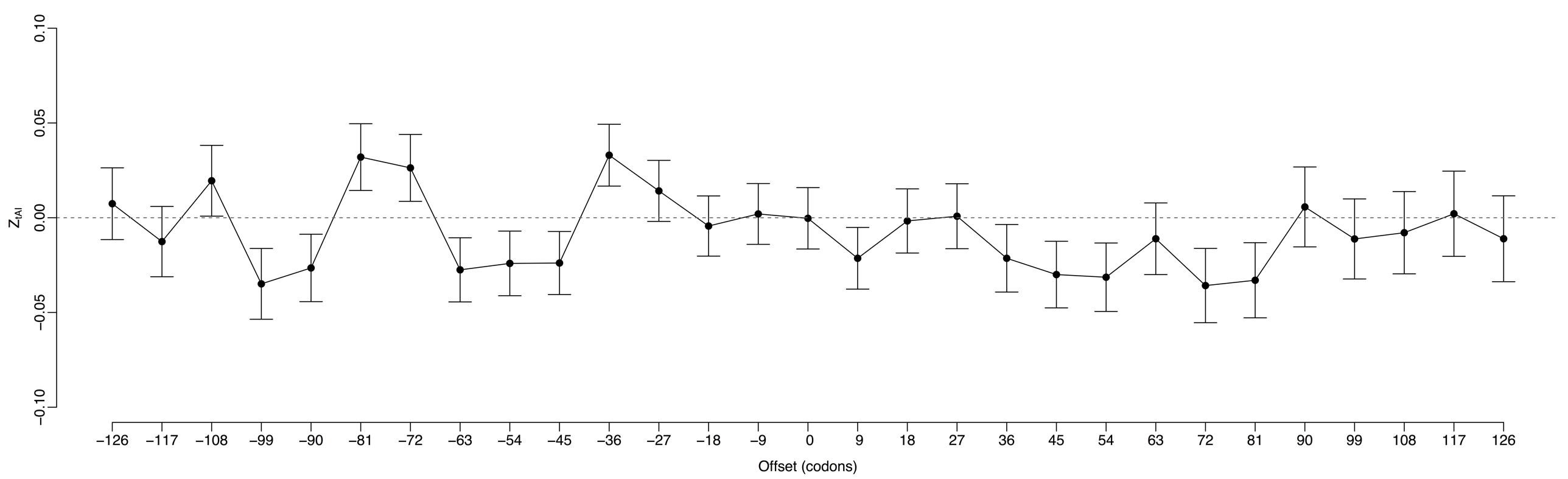

Supplement: Figure S11 — The mean and standard error of of each sliding window near randomized miRNA target region. (TIFF) [file pone.0063403.s011.tiff]
